# Supplementary material for: Projections of the economic burden of care for individuals with dementia in mainland China from 2010 to 2050
Source: PLoS One. 2022 Feb 3;17(2):e0263077. doi: 10.1371/journal.pone.0263077 (PMC8812891; doi:10.1371/journal.pone.0263077)
Supplement: S1 Table — (DOCX) [file pone.0263077.s001.docx]

**S1 Table.** Estimated numbers of people aged 60 years and above and people with dementia in China from 2010 to 2015

|  | 60-64 | 65-69 | 70-74 | 75-79 | ≥80 | Total | Total prevalence  (≥ 60) (%) |
| --- | --- | --- | --- | --- | --- | --- | --- |
| Age-specific prevalence (%) | 1.5 | 2.8 | 3.3 | 5.7 | 15.3 |  |  |
| 2010 population (in thousands) | 58,271.1 | 41,383.4 | 33,394.3 | 23,228.7 | 19,632.2 | 175,909.8 |  |
| Cases (in thousands) | 891.0 | 1,164.4 | 1,117.5 | 1,333.6 | 3,001.0 | 7,507.4 | 4.2 |
| 2015 population (in thousands) | 81,506.1 | 53,652.7 | 35,584.9 | 25,657.8 | 24,316.7 | 220,718.2 |  |
| Cases (in thousands) | 1,246.3 | 1,509.6 | 1,190.8 | 1,473.0 | 3,717.0 | 9,136.7 | 4.1 |
| 2020 population (in thousands) | 78,998.2 | 75,496.5 | 46,651.4 | 27,812.5 | 28,828.8 | 257,787.3 |  |
| Cases (in thousands) | 1,207.9 | 2,124.2 | 1,561.2 | 1,596.7 | 4,406.7 | 10,896.7 | 4.2 |
| 2025 population (in thousands) | 98,465.2 | 73,590.1 | 66,314.2 | 37,123.7 | 33,131.0 | 308,624.2 |  |
| Cases (in thousands) | 1,505.6 | 2,070.5 | 2,219.2 | 2,131.3 | 5,064.3 | 12,990.9 | 4.2 |
| 2030 population (in thousands) | 118,065.9 | 92,206.4 | 65,277.5 | 53,621.5 | 42,567.2 | 371,738.5 |  |
| Cases (in thousands) | 1,805.3 | 2,594.3 | 2,184.5 | 3,078.4 | 6,506.7 | 16,169.2 | 4.4 |
| 2035 population (in thousands) | 112,213.6 | 111,112.3 | 82,525.7 | 53,632.2 | 60,712.2 | 420,196.1 |  |
| Cases (in thousands) | 1,715.8 | 3,126.3 | 2,761.7 | 3,079.0 | 9,280.3 | 19,963.1 | 4.8 |
| 2040 population (in thousands) | 91,308.0 | 106,098.3 | 100,289.6 | 68,765.6 | 72,317.1 | 438,778.7 |  |
| Cases (in thousands) | 1,396.1 | 2,985.2 | 3,356.1 | 3,947.8 | 11,054.2 | 22,739.5 | 5.2 |
| 2045 population (in thousands) | 96,836.9 | 86,737.4 | 96,546.1 | 84,713.0 | 90,785.8 | 455,619.2 |  |
| Cases (in thousands) | 1,480.7 | 2,440.4 | 3,230.9 | 4,863.4 | 13,877.3 | 25,892.6 | 5.7 |
| 2050 population (in thousands) | 122,148.8 | 92,379.9 | 79,559.7 | 82,606.3 | 115,246.3 | 491,941.1 |  |
| Cases (in thousands) | 1,867.7 | 2,599.2 | 2,662.4 | 4,742.4 | 17,616.2 | 29,488.0 | 6.0 |
