# Supplementary material for: Projections of the economic burden of care for individuals with dementia in mainland China from 2010 to 2050
Source: PLoS One. 2022 Feb 3;17(2):e0263077. doi: 10.1371/journal.pone.0263077 (PMC8812891; doi:10.1371/journal.pone.0263077)
Supplement: S2 Table — (DOCX) [file pone.0263077.s002.docx]

**S2 Table.** Annual cost of care for individuals with dementia in China from 2010 to 2050, differentiated by methods measuring indirect costs without discounting

|  | 2010 | 2015 | 2020 | 2025 | 2030 | 2035 | 2040 | 2045 | 2050 |
| --- | --- | --- | --- | --- | --- | --- | --- | --- | --- |
| Direct medical costs | 2.0 | 2.4 | 2.9 | 3.4 | 4.2 | 5.2 | 5.9 | 6.8 | 7.7 |
| Direct non-medical costs | 1.8 | 2.3 | 2.7 | 3.2 | 4.1 | 5.0 | 5.6 | 6.4 | 7.3 |
| Formal caregivers | 1.5 | 1.8 | 2.2 | 2.6 | 3.3 | 4.1 | 4.7 | 5.3 | 6.1 |
| Transportation | 0.2 | 0.3 | 0.4 | 0.4 | 0.5 | 0.7 | 0.7 | 0.8 | 0.9 |
| Special equipment | 0.1 | 0.1 | 0.1 | 0.1 | 0.2 | 0.2 | 0.2 | 0.3 | 0.3 |
| Indirect costs |  |  |  |  |  |  |  |  |  |
| Informal caregivers  (opportunity cost method) | 19.0 | 23.2 | 27.6 | 32.9 | 41.0 | 50.6 | 57.6 | 65.6 | 74.7 |
| Informal caregivers  (proxy method) | 22.5 | 27.4 | 32.7 | 39.0 | 48.5 | 59.9 | 68.3 | 77.7 | 88.5 |
| Total (opportunity cost method) | 22.8 | 27.8 | 33.1 | 39.5 | 49.2 | 60.8 | 69.2 | 78.8 | 89.5 |
| Total (proxy method) | 26.4 | 32.1 | 38.2 | 45.6 | 56.8 | 70.1 | 79.8 | 90.9 | 103.6 |
