# Supplementary material for: Projections of the economic burden of care for individuals with dementia in mainland China from 2010 to 2050
Source: PLoS One. 2022 Feb 3;17(2):e0263077. doi: 10.1371/journal.pone.0263077 (PMC8812891; doi:10.1371/journal.pone.0263077)
Supplement: S3 Table — NOTE: Data for national GDP refer to the projections of Goldman Sachs. *Cost estimated without consideration as to whether patients have been diagnosed or not. (DOCX) [file pone.0263077.s003.docx]

**S3 Table.** Annual cost of care for individuals with dementia in China from 2010 to 2050, differentiated by methods measuring indirect costs without discounting

| Cost of dementia, US$ billion | 2010 | 2010^*^ | 2015 | 2015^*^ | 2020 | 2020^*^ | 2025 | 2025^*^ | 2030 | 2030^*^ | 2035 | 2035^*^ | 2040 | 2040^*^ | 2045 | 2045^*^ | 2050 | 2050^*^ |
| --- | --- | --- | --- | --- | --- | --- | --- | --- | --- | --- | --- | --- | --- | --- | --- | --- | --- | --- |
| Direct medical costs | 2.0 | 25.0 | 2.4 | 30.0 | 2.9 | 36.3 | 3.4 | 42.5 | 4.2 | 52.5 | 5.2 | 65.0 | 5.9 | 73.8 | 6.8 | 85.0 | 7.7 | 96.3 |
| Direct non-medical costs | 1.8 | 24.5 | 2.3 | 31.3 | 2.7 | 36.8 | 3.2 | 43.6 | 4.1 | 55.8 | 5.0 | 68.1 | 5.6 | 76.2 | 6.4 | 87.1 | 7.3 | 99.4 |
| Formal caregivers | 1.5 | 20.4 | 1.8 | 24.5 | 2.2 | 29.9 | 2.6 | 35.4 | 3.3 | 44.9 | 4.1 | 55.8 | 4.7 | 63.9 | 5.3 | 72.1 | 6.1 | 83.0 |
| Transportation | 0.2 | 3.1 | 0.3 | 4.7 | 0.4 | 6.2 | 0.4 | 6.2 | 0.5 | 7.8 | 0.7 | 10.9 | 0.7 | 10.9 | 0.8 | 12.4 | 0.9 | 14.0 |
| Special equipment | 0.1 | 1.0 | 0.1 | 1.0 | 0.1 | 1.0 | 0.1 | 1.0 | 0.2 | 2.0 | 0.2 | 2.0 | 0.2 | 2.0 | 0.3 | 3.0 | 0.3 | 3.0 |
| Indirect costs |  |  |  |  |  |  |  |  |  |  |  |  |  |  |  |  |  |  |
| Informal caregivers  (opportunity cost method) | 19.0 | 63.0 | 23.2 | 76.9 | 27.6 | 91.5 | 32.9 | 109.1 | 41.0 | 135.9 | 50.6 | 167.8 | 57.6 | 191.0 | 65.6 | 217.5 | 74.7 | 247.7 |
| Informal caregivers  (proxy method) | 22.5 | 74.6 | 27.4 | 90.8 | 32.7 | 108.4 | 39.0 | 129.3 | 48.5 | 160.8 | 59.9 | 198.6 | 68.3 | 226.5 | 77.7 | 257.6 | 88.5 | 293.4 |
| Total  (opportunity cost method) | 22.8 | 112.5 | 27.8 | 137.2 | 33.1 | 163.3 | 39.5 | 194.9 | 49.2 | 242.8 | 60.8 | 300.0 | 69.2 | 341.4 | 78.8 | 388.8 | 89.5 | 441.6 |
| Total  (proxy method) | 26.4 | 124.1 | 32.1 | 150.9 | 38.2 | 179.6 | 45.6 | 214.4 | 56.8 | 267.0 | 70.1 | 329.5 | 79.8 | 375.1 | 90.9 | 427.3 | 103.6 | 487.0 |

NOTE: Data for national GDP refer to the projections of Goldman Sachs.

*Cost estimated without consideration as to whether patients have been diagnosed or not.
