# Supplementary material for: Projections of the economic burden of care for individuals with dementia in mainland China from 2010 to 2050
Source: PLoS One. 2022 Feb 3;17(2):e0263077. doi: 10.1371/journal.pone.0263077 (PMC8812891; doi:10.1371/journal.pone.0263077)
Supplement: S4 Table — (DOCX) [file pone.0263077.s004.docx]

**S4 Table.** Sensitivity analysis by the opportunity cost method

| Annual cost of dementia, US$ billion (%) | 2010 | 2015 | 2020 | 2025 | 2030 | 2035 | 2040 | 2045 | 2050 |
| --- | --- | --- | --- | --- | --- | --- | --- | --- | --- |
| Base option | 22.9 | 33.2 | 47.4 | 67.5 | 100.4 | 148.1 | 201.6 | 274.3 | 373.3 |
| Undetected rate from 93.1% to 73.1% | 28.4 (+24.3) | 41.3 (+24.3) | 58.9 (+24.3) | 83.8 (+24.3) | 124.7 (+24.3) | 184 (+24.3) | 250.4 (+24.3) | 340.8 (+24.3) | 463.8 (+24.3) |
| 5% annual increase of medical costs | 22.9 (0) | 34 (+2.4) | 49.9 (+5.4) | 73.7 (+9.3) | 114.6 (+14.2) | 178.4 (+20.5) | 259.1 (+28.5) | 380.7 (+38.8) | 567.0 (+51.9) |
| Proportion of informal care from 30.2% to 93.1% | 58.0 (+153.7) | 84.3 (+153.7) | 120.2 (+153.7) | 171.2 (+153.7) | 254.7 (+153.7) | 375.7 (+153.7) | 511.5 (+153.7) | 695.9 (+153.7) | 947.1 (+153.7) |
| Proportion of formal care from 4.9% to 7.6% | 22.3 (-2.4) | 32.4 (-2.4) | 46.2 (-2.4) | 65.9 (-2.4) | 98.0 (-2.4) | 144.6 (-2.4) | 196.8 (-2.4) | 267.8 (-2.4) | 364.4 (-2.4) |
| Wages of informal caregivers based on national mean salary | 36.9 (+61.5) | 53.7 (+61.5) | 76.5 (+61.5) | 109 (+61.5) | 162.1 (+61.5) | 239.2 (+61.5) | 325.6 (+61.5) | 443.0 (+61.5) | 602.9 (+61.5) |
| Daily informal care time 15.4 hours to 6.3 hours | 11.6 (-49.2) | 16.9 (-49.2) | 24.1 (-49.2) | 34.3 (-49.2) | 51.0 (-49.2) | 75.2 (-49.2) | 102.3 (-49.2) | 139.3 (-49.2) | 189.5 (-49.2) |
| Discount rate of 3% | 22.8 (0) | 32.4 (-2.6) | 45.0 (-5.1) | 62.4 (-7.5) | 90.5 (-9.8) | 130.1 (-12.1) | 172.6 (-14.4) | 228.8 (-16.6) | 303.5 (-18.7) |
| Discount rate of 5% | 22.9 (+0.1) | 36 (+8.2) | 55.4 (+17.0) | 85.4 (+26.6) | 137.4 (+36.9) | 219.2 (+48.0) | 322.7 (+60.1) | 474.9 (+73.2) | 699.0 (+87.3) |
| Prevalence from systematic review in 2010 | 28.4 (+24.5) | 41.0 (+23.3) | 58.9 (+24.3) | 86.0 (+27.4) | 127.1 (+26.6) | 186.4 (+25.9) | 266.4 (+32.2) | 371.3 (+35.4) | 506.2 (+35.6) |
