# Supplementary material for: Projections of the economic burden of care for individuals with dementia in mainland China from 2010 to 2050
Source: PLoS One. 2022 Feb 3;17(2):e0263077. doi: 10.1371/journal.pone.0263077 (PMC8812891; doi:10.1371/journal.pone.0263077)
Supplement: S5 Table — (DOCX) [file pone.0263077.s005.docx]

**S5 Table.** Sensitivity analysis by the proxy method

| Annual cost of dementia, US$ billion (%) | 2010 | 2015 | 2020 | 2025 | 2030 | 2035 | 2040 | 2045 | 2050 |
| --- | --- | --- | --- | --- | --- | --- | --- | --- | --- |
| Base option | 26.4 | 38.3 | 54.6 | 77.8 | 115.8 | 170.8 | 232.5 | 316.4 | 430.6 |
| Undetected rate from 93.1% to 73.1% | 31.9 (+21.0) | 46.4 (+21.0) | 66.1 (+21.0) | 94.2 (+21.0) | 140.1 (+21.0) | 206.7 (+21.0) | 281.4 (+21.0) | 382.9 (+21.0) | 521.1 (+21.0) |
| 5% annual increase of medical costs | 26.4 (0) | 39.1 (+2.1) | 57.2 (+4.7) | 84.1 (+8.0) | 130 (+12.3) | 201.2 (+17.8) | 290.1 (+24.7) | 422.8 (+33.6) | 624.3 (+45.0) |
| Proportion of informal care from 30.2% to 93.1% | 68 (+157.9) | 98.9 (+157.9) | 140.9 (+157.9) | 200.7 (+157.9) | 298.6 (+157.9) | 440.5 (+157.9) | 599.6 (+157.9) | 815.9 (+157.9) | 1,110.3 (+157.9) |
| Proportion of formal care from 4.9% to 7.6% | 25.8 (-2.1) | 37.5 (-2.1) | 53.5 (-2.1) | 76.3 (-2.1) | 113.4 (-2.1) | 167.3 (-2.1) | 227.8 (-2.1) | 309.9 (-2.1) | 421.8 (-2.1) |
| Daily informal care time from 15.4 hours to 6.3 hours | 13 (-50.5) | 19 (-50.5) | 27 (-50.5) | 38.5 (-50.5) | 57.3 (-50.5) | 84.5 (-50.5) | 115 (-50.5) | 156.5 (-50.5) | 213 (-50.5) |
| Discount rate of 3% | 26.4 (0) | 37.4 (-2.6) | 51.9 (-5.1) | 72 (-7.5) | 104.4 (-9.8) | 150.1 (-12.1) | 199.1 (-14.4) | 264 (-16.6) | 350 (-18.7) |
| Discount rate of 5% | 26.4 (+0.1) | 41.5 (+8.2) | 63.9 (+17.0) | 98.5 (+26.6) | 158.5 (+36.9) | 252.9 (+48.0) | 372.3 (+60.1) | 547.8（+73.1) | 806.3 (+87.2) |
| Prevalence from systematic review in 2010 | 32.8 (+24.5) | 47.3 (+23.3) | 67.9 (+24.3) | 99.2 (+27.4) | 146.6 (+26.6) | 215.1 (+25.9) | 307.4 (+32.2) | 428.3 (+35.4) | 584 (+35.6) |
