# Supplementary material for: Projections of the economic burden of care for individuals with dementia in mainland China from 2010 to 2050
Source: PLoS One. 2022 Feb 3;17(2):e0263077. doi: 10.1371/journal.pone.0263077 (PMC8812891; doi:10.1371/journal.pone.0263077)
Supplement: S2 Fig — (DOCX) [file pone.0263077.s007.docx]

**S2 Fig.** The annual cost of care for individuals with dementia in China by the proxy method.
